# Vulnerable patients going to court: a psychiatrist's guide to special measures

**DOI:** 10.1192/pb.bp.115.051508

**Published:** 2016-08

**Authors:** Penny Cooper, Janet Grace

**Affiliations:** 1 Institute for Criminal Policy Research, Birkbeck, University of London; 2 39 Essex Chambers, London; 3 Northumberland, Tyne and Wear NHS Foundation Trust, Newcastle; 4 KITE Newcastle University Business School

## Abstract

There have been significant changes to how vulnerable people are treated in the court system, including the introduction of special measures to support people both as witness and as accused. This paper summarises the use of special measures and their application to people with mental health diagnoses or cognitive impairment.

It is not uncommon for psychiatric patients to be involved in court proceedings and as many psychiatrists know (some from first-hand experience) going to court can be a stressful event. In addition, mental illness, neurocognitive impairment or developmental disorder can create a barrier to access to giving evidence or to being able to take part in a trial. In recognition of this, major changes in the judicial approach have been made, including the use of ‘special measures’ for certain witnesses. This article summarises the latest judicial approaches and considers their clinical applications.

## The criminal justice system leads the way

Over the past 10 years the criminal justice system has led the way in making adjustments for vulnerable witnesses and defendants. Every judge's primary duty is to ensure that there is a fair trial and judges in criminal cases have at their disposal a range of special measures for ‘vulnerable’ and ‘intimidated’ witnesses.

Any person under 18 years or whose ability to take part in a trial, either as a defendant or a witness, is affected by a mental or physical disorder or impairment of their intellectual or social functioning is potentially vulnerable and eligible for special measures. The person's views must be taken into account. Even if no party applies for them, the judge can decide that special measures should be used.

Witness special measures (note the legislation makes it clear these are not for the defendant) are set out in the Youth Justice and Criminal Evidence Act 1999 (Sections 23–30).

- ‘Screening witness from accused’: a curtain (usually) can be drawn around the witness box so that the vulnerable (or intimidated) witness can enter the court and give evidence unseen by the defendant. The witness can, however, be seen by the judge, jury and the advocates.
- ‘Evidence by live link’ (also known as TV link): a witness can give evidence over the closed-circuit television usually linked to another room in the court building. Sometimes witnesses give evidence by live link from a remote location such as a hospital or a care home. A judge may also direct that a witness ‘supporter’ can be present with the witness while they give evidence.
- ‘Evidence given in private’: the judge can order that the public gallery is cleared.
- ‘Removal of wigs and gowns’: not all vulnerable witnesses prefer this. Some prefer judges and advocates to wear their easily recognisable ‘uniform’.
- ‘Video-recorded evidence in chief’: the police usually interview vulnerable witness in a video suite and the DVD of that interview (edited down if necessary) can be played as an alternative to the witness giving their account in the witness box.
- ‘Video-recorded cross-examination or re-examination’: so far this has only been trialled in three Crown Courts but the anecdotal feedback thus far has been mostly positive. Under the pilot scheme witnesses do not have to wait for the trial to give their evidence ‘live’ but instead go to court (often within weeks of the defendant being charged) and are recorded being questioned by the defence team. The tape of the questioning is then played in place of them attending at the trial for cross-examination.
- ‘Examination of witness through intermediary’: the intermediary role was first introduced in 2003 and is defined in the legislation as something akin to an interpreter; however, intermediary practice has evolved greatly since those early days such that the role is now much broader than first envisaged.^1^ The police or Crown Prosecution Service can request the services of an intermediary^2^ (a communication specialist) who will assess the witness, then advise the court and the advocates what steps should be put in place to ensure that the witness understands the questions and the answers that they give are understood. The intermediary can monitor communication in court (usually sitting alongside the witness) and, for example, intervene during cross-examination if the question has not been put in a way that the witness can deal with.
- ‘Aids to communication’: there is no set definition of communication aid so that could be anything from using maps and photographs to a computerised system for the witness to spell out their answers.^3^

Special measures can be, and frequently are, used in combination.

## Defendants as well as witnesses

The Youth Justice and Criminal Evidence Act 1999 specifically excluded the accused from special measures. However, the Police and Justice Act 2006 allows vulnerable defendants to give evidence by live link.

Section 104 of the Coroners and Justice Act 2009 allows for certain vulnerable accused individuals to give oral evidence at trial with the assistance of an intermediary. That section has not yet been implemented, and registered intermediaries are therefore not available for defendants. In trials in which a defendant has communication or other difficulties, the judge has a duty to ensure that there is a fair trial by making such adaptations to the trial process as are in the interests of justice to ensure the defendant's effective participation. If it is deemed to be in the interests of justice the judge will direct an intermediary for the vulnerable defendant either for the whole trial or, less usually, only if the defendant gives evidence.

## Best practice for court

The Criminal Practice Directions,^4^ guidance that clarifies the practical application of the law, made clear and specific recommendations about vulnerable witnesses and defendants.

‘ … many other people giving evidence in a criminal case, whether as a witness or defendant, may require assistance: the court is required to take “every reasonable step” to encourage and facilitate the attendance of witnesses and to facilitate the participation of any person, including the defendant (Rule 3.8(4)(a) and (b)). This includes enabling a witness or defendant to give their best evidence, and enabling a defendant to comprehend the proceedings and engage fully with his or her defence’ (CPD, 3D.2).

The responsibility for identifying vulnerability in witness or defendant is not identified in the Criminal Practice Directions and lies with those professionals who interact with the person at various steps within the process, namely police, solicitors and barristers. If anyone is uncertain about the witness's or defendant's vulnerability, they may request an expert psychiatric report and/or an intermediary assessment.

Frequently psychiatrists will be asked to assess a defendant's fitness to plead and one possible conclusion is that the defendant could benefit from the assistance of an intermediary. Psychiatrists should not express the opinion that an intermediary will ensure the defendant is fit to plead, but instead the defendant might be fit to plead if assisted by an intermediary. The impact of this special measure on the defendant's fitness to plead will not be known unless and until the intermediary has assessed the defendant's communication needs and abilities and determined how, if at all, the defendant will benefit from their assistance at trial. The Law Commission^5^ is currently undertaking a review of the law in relation to fitness to plead, including the provision of intermediaries for vulnerable defendants, and by the end of 2015 it is likely there will be a proposal for new legislation.

**Box 1** Case example 1

L.M. is a 34-year-old man with autism and mild intellectual disability who lives in long-term residential care. He copes badly with change, has agoraphobia and becomes incoherent and anxious when faced with stressful situations. He has a variety of reassuring rituals, including tapping, wearing a baseball cap low over his face and at times turning his back to the person he is talking to. L.M. was a witness to a series of physical abuse to his peer in a previous residential unit. When seen by the consultant psychiatrist he was able to give an internally consistent account of what had happened. When the psychiatrist spoke to the court, L.M. was allowed to give his evidence via a video link, wearing his baseball cap, with an allowance for breaks if needed.

**Box 2** Case example 2

J.K. is a 57-year-old man with stroke-related memory problems. He has a Mini-Mental State Examination^6^ score of 23/30 and has difficulty retaining information. In day-to-day life, he is reliant on support from statutory services and his family. He uses notes and a diary and will often need information repeated to be able to retain it. He is accused of theft from a shop. He is keen to go to court but is worried about being able to follow the trial. Following advice by his treating psychologist, J.K. is offered an intermediary to help him note down and retain information with breaks scheduled into the court process to allow him to review his notes and discuss events with his legal team with the assistance of the intermediary.

Information from the treating team about the vulnerable witness or defendant may also be requested in the context of a court case arising within the period of treatment by secondary or tertiary mental health services. Two clinical scenarios are described in Box 1 and Box 2.

Judges have a wide discretion to put in place special measures to ensure a fair trial. They are not limited to the special measures set out in the legislation.^7^ Any adjustments to the traditional court process will of course be specific to the witness or defendant but may include such diverse interventions as:

- requiring everyone throughout the trial to slow down and use plain and simple language
- allowing the defendant to sit next to counsel (as opposed to the usual place in the dock)
- scheduling extraordinary breaks throughout the trial
- use of a stress toy or similar for state management (e.g. use of a sponge stress toy for the defendant to squeeze while in the dock with dock officer agreement)
- allowing the defendant to wear certain clothing (e.g. a baseball cap in court).

## Ground rules hearings

An application to the judge for special measures should be made at the earliest opportunity. Closer to the trial the parameters for the fair treatment and questioning of the vulnerable person must be discussed by the trial judge, advocates and intermediary (if there is one) at what is called a ‘ground rules hearing’ (Criminal Procedure (Amendment) Rules 2015). If the ground rules that are being discussed are based on the psychiatrist's recommendations, the psychiatrist may be asked to attend to assist the court at the ground rules hearing. As well as discussing the practicalities for implementing special measures at this hearing, the judge may require the advocates to go through their cross-examination questions with the intermediary or the psychiatrist. ‘Advocates must adapt to the witness, not the other way round’, said the vice-president of the Court of Appeal (Criminal Division), Lady Justice Hallett DBE in *R v Lubemba* [2014].^8^ In that judgment Lady Hallett also endorsed The Advocate's Gateway (www.theadvocatesgateway.org), which provides free, research-based guidance for advocates on questioning vulnerable people and working with witnesses and defendants with mental health disorder.

## Other courts follow suit

Special measures are not simply for criminal cases. Other courts, although lagging far behind the criminal justice system with respect to vulnerable witnesses, are now working hard to catch up and are adopting the vocabulary and practices of the criminal justice system. For instance, in individual cases family civil and mental health tribunal judges have adopted the ground rules approach and directed special measures.^9,10^ New rules and practice directions about child witnesses and vulnerable parties in the family courts are due to be in place by the beginning of 2016. Whether your patient is a witness in a criminal trial, a defendant charged with a criminal offence, a parent in a family court dispute about their child, a claimant in a personal injury case, etc., they are entitled to be considered for special measures. The psychiatrist's advice on the most appropriate adjustments can play a key role in ensuring a fair trial, as illustrated in the final case study in Box 3.

**Box 3** Case example 3

I.J. was a patient taking a significant amount of medication to control psychiatric symptoms. Her ability to give coherent testimony was much improved in the afternoon when her medication had the chance to start working and her mental state was most stable. The court schedule was adjusted so that she gave her evidence in the afternoons.

## Conclusions

Recent significant changes to court practice are designed to facilitate fair hearings for all. An awareness of special measures and this fast developing area of law will allow psychiatrists to ensure that patients who are required to attend court can participate to the best of their ability.

To view a free training film demonstrating the use of an intermediary and other special measures, go to www.theadvocatesgateway.org/a-question-of-practice
